# Hyperuricemia and Associated Factors in Children with Chronic Kidney Disease: A Cross-Sectional Study

**DOI:** 10.3390/children9010006

**Published:** 2021-12-23

**Authors:** Jie Xu, Lingxiao Tong, Jianhua Mao

**Affiliations:** Department of Nephrology, The Children’s Hospital, Zhejiang University School of Medicine, National Clinical Research Center for Child Health, #57 Zhugan Lane, Hangzhou 310003, China; 11918401@zju.edu.cn (J.X.); 11718193@zju.edu.cn (L.T.)

**Keywords:** serum uric acid, hyperuricemia, chronic kidney disease, children

## Abstract

Background: Hyperuricemia is increasingly recognized as a risk factor for chronic kidney disease (CKD) just in adults. The purpose of this study was to investigate the clinical characteristics of hyperuricemia and its associated factors in Chinese children with CKD at a single center. Methods: A cross-sectional study of 170 CKD children collected from the Department of Nephrology, The Zhejiang University Children’s Hospital was conducted. The clinical data, including anthropometric data, blood pressure measurements, and biochemical parameters, were recorded and analyzed retrospectively. The factors associated with hyperuricemia in CKD children were evaluated by Pearson and Spearman correlation analysis and multiple logistic regression analysis. Results: The mean age was 9.79 ± 4.10 years, and 72 (42.35%) were girls. Higher blood urea nitrogen (BUN), serum creatinine, cystatin C, D-dimer, lower hemoglobin, albumin, and estimated glomerular filtration rate (eGFR) were significantly associated with higher serum uric acid (SUA). In multiple logistic regression analysis, anemia and higher BUN were both positively associated factors, whereas eGFR ≥ 90 mL/min/1.73 m^2^ was a negatively associated factor for subjects with SUA ≥ 390 µmol/L (6.5 mg/dL). Conclusions: SUA was significantly associated with kidney risk factors in CKD children. Monitoring and controlling SUA, Hb, BUN, and Scr levels in CKD children may help to prevent the progression of CKD.

## 1. Introduction

Hyperuricemia is a chronic purine metabolic disorder mainly because of excessive production of uric acid and/or decreased renal excretion. Recently, the prevalence of hyperuricemia has markedly increased among children worldwide [1,2,3]. Many clinical and epidemiological studies have suggested that increased serum uric acid (SUA) levels may be closely associated with metabolic syndrome, chronic kidney disease (CKD), and cardiovascular disease [4,5,6,7,8,9]. Indeed, hyperuricemia in children has become a major public health issue and is currently gaining more and more attention.

CKD causes a substantial burden to individuals, families, and health care systems due to the reduced quality of life, dialysis, and even kidney transplantation [10]. Hyperuricemia and CKD probably influence one another in many ways, depending on multiple mechanisms. Impaired kidney function may contribute to hyperuricemia because of the decreased renal excretion [11]. On the other hand, hyperuricemia plays an important role in the development and progression of CKD due to several factors, such as vascular smooth cell proliferation, endothelial dysfunction, impaired endothelial nitric oxide production, and inflammation [4,12,13,14]. However, what factors may be associated with hyperuricemia in CKD children remains uncertain.

The aims of the cross-sectional study were to provide reliable information on the clinical characteristics of hyperuricemia in children with CKD from a pediatric nephrology department and to further evaluate the association between hyperuricemia and its associated factors in Chinese CKD children.

## 2. Materials and Methods

### 2.1. Study Sample

We collected the records of 170 children and adolescents diagnosed with CKD hospitalized at Zhejiang University Children’s Hospital between January 2013 and September 2020. The patients had a mean age of 9.79 ± 4.10 years, and 72 (42.35%) were girls. Children who had taken treatments that could affect uric acid metabolism, such as urate-lowering drugs including allopurinol and benzbromarone, were excluded. Subjects with a history of severe infection, cardiovascular disease, liver injury, kidney transplantation, dialysis, tumor, and malignant hematological disease were excluded from the study. The ethics committee of Zhejiang University Children’s Hospital approved the study.

### 2.2. Study Measures and Laboratory Methods

Participants provided clinical data, including anthropometric data, blood pressure (BP) measurements, biochemical parameters, and prescription medications. BP and anthropometric data, including height and weight, were undertaken by the professional nurses. The biochemical parameters including hemoglobin (Hb), albumin (Alb), total cholesterol (TC), triglycerides (TG), blood urea nitrogen (BUN), serum uric acid (SUA), cystatin C (Cys C), serum creatinine (Scr), fibrinogen (Fib) and D-Dimer (DD) were measured. The estimated glomerular filtration rate (eGFR) was calculated using the Schwartz formula [15] as follows: eGFR (mL/ min/1.73 m^2^) = *k* × *L*/Scr. The value of *k* is a constant varying with age and sex, *L* stands for height (cm), and Scr stands for serum creatinine (μmol/L). Urine was collected for 24 h, and 24 h urine protein quantity (24HUPr) was measured. The professional doctors collected the medical history of participants, including etiological factors, prescription drugs, and family history.

Obesity was estimated by body mass index (BMI) more than the 95th percentile based on age and sex [16]. Hypertension was defined as average measured systolic blood pressure (SBP) and/or diastolic blood pressure (DBP) ≥95th percentile according to sex, age, and height percentiles [17]. Anemia in children with CKD was diagnosed if Hb concentration is <110 g/L in children 0.5–5 years, <115 g/L in children 5–12 years, and <120 g/L in children 12–15 years [18]. CKD was defined as abnormalities of renal structure or function lasting for at least 3 months and can be classified into 5 stages based on the Kidney Disease: Improving Global Outcomes (KDIGO) guidelines [19]. Congenital anomalies of the kidney and urinary tract (CAKUT) included obstructive uropathy, aplasia/hypoplasia/dysplasia, and reflux nephropathy [20]. Those whose length or height *z*-score was 2 SD score or more below the mean height based on age, sex, and population were diagnosed with short stature [21]. Those renal osteopathy patients were diagnosed with chronic kidney disease-mineral and bone disorder (CKD-MBD), including renal osteodystrophy and extra-skeletal calcification due to the abnormalities of bone mineral metabolism [18]. Elevated TC was defined as TC was ≥200 mg/dL in children and adolescents. Elevated TG was defined as TG was ≥100 mg/dL in children 0–9 years and ≥130 mg/dL in children 10–19 years [22]. In children, SUA levels increased with age, and there were no universally accepted clinical diagnostic criteria of hyperuricemia. In adults, hyperuricemia was defined that SUA levels were ≥416.0 µmol/L (7.0 mg/dL) in male and ≥357.0 µmol/L (6.0 mg/dL) in female [23]. The China National Survey of Chronic Kidney Disease reported that SUA levels of adults with CKD were 387.0 ± 129.5 µmol/L [24]. What is more, studies indicated that SUA levels at 6.3–6.5 mg/dL contributed to the progression of CKD [25,26]. So, in this study, elevated SUA was defined as ≥390 µmol/L (6.5 mg/dL), also representing the approximate IQR of the distribution.

### 2.3. Statistical Analysis

The continuous data were presented as mean ± SD, and categorical data were presented as numbers and percentages. In the case of nonparametric distribution, data (including BUN, Scr, and 24HUPr) were presented as a median (interquartile range, IQR). Student’s *t*-tests and Mann–Whitney U test were used to compare continuous characteristics by sex at baseline. Categorical characteristics were compared by χ^2^ tests. Characteristics with a skewed distribution (including age, BMI, Alb, TC TG, Fib, and DD) were reciprocal-transformed, square-root-transformed, or log-transformed as appropriate. Serum uric acid was categorized as <390 µmol/L (6.5 mg/dL), 390–540 µmol/L (6.5–9 mg/dL), or >540 µmol/L (9 mg/dL) in the cross-sectional analysis, as 390 µmol/L and 540 µmol/L represent the approximate IQR of the distribution. The univariate analysis of variance was used to measure the parametric distribution data among the groups or a Kruskal–Wallis test. For categorical data and proportions, the Bonferroni test was used to compare the difference among groups. The correlations between SUA and the associated factors for CKD were investigated using Pearson and Spearman correlation test. Since SBP, TC, Scr, BUN, Cys C, eGFR, and 24HUPr were not parametrically distributed, we used Spearman correlation analysis. We then used multiple logistic regression analysis to determine the association between potential associated factors and elevated SUA. The results were reported as odds ratios (ORs) with 95% confidence intervals (CIs). Statistical significance for all analyses was set at *p* < 0.05. Statistical analysis was performed with software SPSS 22.0.

## 3. Results

### 3.1. Baseline Characteristics of the Study Population

Totally 170 children were recruited, comprising 98 boys and 72 girls (42.35%), with a mean age of 9.79 ± 4.10 years. General data appeared in Table 1. The mean SUA was 476.96 ± 155.97 µmol/L, and boys had greater SUA levels than girls (497.95 ± 164.45 vs. 448.38 ± 139.70 µmol/L, *p* = 0.04). Boys were taller than girls (132.11 ± 26.80 vs. 126.09 ± 25.30 cm, *p* = 0.031). Median eGFR was 38.26 mL/min/1.73 m^2^. For the etiology of CKD in children, CAKUT had the highest proportion (40.00%, 68/170). In addition, 28.82% (49/170) of CKD participants were diagnosed with glomerular diseases. Then, among the comorbidities of CKD, anemia accounted for 60.59% (103/170), the highest proportion, followed by hypertension 55.89% (95/170). Obesity, short stature, and renal osteopathy accounted for 8.24% (14/170), 24.12% (41/170), and 21.18% (36/170), respectively.

Table 1 reported participants’ characteristics stratified by serum uric acid (<390 µmol/L (6.5 mg/dL; *n* = 52; 30.59%), 390–540 µmol/L (6.5–9.0 mg/dL; *n* = 62; 36.47%), and >540 µmol/L (9.0 mg/dL; *n* = 56; 32.94%)). The participants with SUA >540 µmol/L (9.0 mg/dL) had the highest BUN (*p* < 0.001), Cys C (*p* < 0.001), Scr (*p* < 0.001) and DD (*p* = 0.024) compared to the other two groups. Moreover, the lowest Hb (*p* = 0.002) and eGFR levels were observed in participants with SUA >540 µmol/L (9.0 mg/dL). The participants with SUA ≥ 390 µmol/L (6.5 mg/dL) also had higher incidence of hypertension, anemia and renal osteopathy, compared to those with SUA < 390 µmol/L (6.5 mg/dL). There were similar distributions of age, sex, BMI, BP, TC, TG, Fib, 24HUPr, and the incidence of CAKUT, obesity, hypertension, anemia, short stature, and renal osteopathy among three groups.

### 3.2. Correlations between SUA and Associated Factors in Children with CKD

Table 2 reported the results of the correlation analysis between SUA level and the associated factors for CKD. Pearson and Spearman correlation analysis showed that SUA levels were positively associated with TC (r = 0.161, *p* = 0.036), TG (r = 0.208, *p* = 0.007), Scr (r = 0.515, *p* < 0.001), BUN (r = 0.558, *p* < 0.001), Cys C (r = 0.488, *p* < 0.001), and DD (r = 0.214, *p* = 0.005). SUA levels showed significantly negative correlations with Hb (r = −0.281, *p* < 0.001) and eGFR (r = −0.442, *p* < 0.001).

### 3.3. Relationship between Elevated SUA and Associated Factors

Table 3 showed the results of multiple logistic regression analysis between elevated SUA (≥390 µmol/L, 6.5 mg/dL) and associated factors for CKD. Anemia and higher BUN were both independently associated with a higher risk of elevated SUA (OR: 3.619 (95% CI: 1.322–9.905), OR: 1.113 (95% CI: 1.032–1.200), respectively). In addition, eGFR ≥ 90 mL/min/1.73 m^2^ exhibited an OR of 0.116 (95% CI: 0.025–0.532) for elevated SUA compared with eGFR < 60 mL/min/1.73 m^2^.

## 4. Discussion

The relationship between elevated SUA and metabolic syndrome, cardiovascular diseases, and kidney diseases has been reported in adults and children, but the data are inconsistent, and the results are still controversial. Our study clearly demonstrated associated factors with hyperuricemia in Chinese CKD children for the first time.

Uric acid is a purine metabolite generated in the liver and mostly excreted in the urine [27]. SUA level in children is related to age and sex. There are no universally accepted clinical diagnostic criteria for hyperuricemia in children. During adolescence, boys had higher uric acid levels than similarly aged girls due to lower estrogen levels, increased BMI, and declined fractional excretion of urate [28,29]. In America, hyperuricemia was defined that SUA levels were ≥7.0 mg/dL in males and ≥6.0 mg/dL in females [23]. In Japan, when pediatric patients visited medical clinics, biochemical items, including SUA, in many cases, were measured [2]. The diagnostic criteria for hyperuricemia in Japanese children was based on the cutoffs, which were 5.9 mg/dL for 6–8 years (both genders), 6.1 mg/dL for 9–11 years (both genders), 7.0 mg/dL (males), and 6.2 mg/dL (females) for 12–14 years [30,31]. In the present study, we found that SUA levels were lower in girls, which supported the previous work. Moreover, the percentage of boys with SUA ≥ 390 µmol/L (6.5 mg/dL) was greater than those with SUA < 390 µmol/L (6.5 mg/dL). However, the boy sex with CKD was not significantly associated with elevated SUA (OR: 2.122 (95% CI: 0.917, 4.914)) in the multiple logistic regression analysis. These findings suggested that sex difference was not associated with elevated SUA in CKD children. Together, we estimated that sex differences in SUA levels would continue from adolescence into adulthood, and boys may tolerate higher SUA levels than girls.

This study showed that anemia was positively associated with SUA ≥ 390 µmol/L (6.5 mg/dL). Anemia is a common comorbidity of CKD and has been identified as a risk factor for the progression of CKD [32,33]. Hyperuricemia is prevalent in sickle cell anemia (SCA) patients [34,35,36]. In cross-sectional studies of children with SCA, those with hyperuricemia had a lower eGFR than those without hyperuricemia [34,35]. Additionally, a prospective study reported that the eGFR of SCA children with hyperuricemia declined more quickly during adolescence [36]. Hyperuricemia and anemia are both independent risk factors for CKD. However, the relationship between hyperuricemia and anemia in CKD children has not been adequately studied. Additional prospective studies are needed to determine the relationship between hyperuricemia and anemia in the setting and the development of CKD in children.

Studies have suggested that elevated SUA levels were associated with hypertension in CKD [4,37,38,39,40]. Animal experiments have reported that elevated SUA levels can activate the renin-angiotensin-aldosterone system and the sympathetic nervous system, mediate endothelial dysfunction, and increase oxidative stress and inflammation, resulting in vasoconstriction and hypertension [4,39,40]. A large study of 2373 high school people found that SUA level had become the most important SBP determinant [38]. A small (*n* = 63) study of pediatric hemodialysis patients found that pre-treatment SBP was associated with a higher SUA level [37]. In contrast, we found that hypertension was not associated with elevated SUA in CKD children. It was likely that blood pressure had a weaker relationship with SUA in children, possibly related to sex hormones, growth, and other variables.

Numerous studies have noted that elevated SUA level is associated with an increased risk of the progression of CKD in the general population [29,41,42,43]. However, not all findings have been positive, and related studies in children are still limited. In a study of 678 participants aged ≤15.6 years, researchers found that those with SUA >7.5 mg/dL had faster progression to >30% decline in GFR [8]. Moreover, a randomized clinical trial including 113 participants showed that urate-lowering treatment with allopurinol may decrease the rate of decline in kidney function [44]. However, a recent randomized clinical trial of 363 adults with stage 3–4 CKD found that allopurinol did not slow the progression of CKD compared with placebo [45]. In another randomized clinical trial of 70 children with CKD stages 1–3, allopurinol can improve renal function [46]. Our study showed that eGFR ≥ 90 mL/min/1.73 m^2^ was a protective factor for elevated SUA in CKD children. We also observed that a higher BUN level was positively associated with SUA ≥ 390 µmol/L (6.5 mg/dL). Those would suggest that early detection and early treatment of hyperuricemia for CKD children might retard the progression of CKD but need to be explored further.

Our study has several limitations. Firstly, this study was a cross-sectional study based on information from the hospital, so it was hard to infer a causal relationship between exposure and outcomes. Moreover, these data from the hospital from 2013 to 2020 were a little unreliable because of the changes of the devices, the conditions, and the methods over time. Furthermore, we used eGFR estimated by the Schwartz formula rather than direct measurements to evaluate kidney function [15]. The Schwartz formula, which was developed in American children, might be unsuitable for Chinese children to estimate the GFR. Finally, patients who are hospitalized at this hospital might have more severe clinical symptoms than outpatients, and the data from those patients might overestimate associated factors.

## 5. Conclusions

In conclusion, we demonstrate that SUA was significantly associated with anemia, BUN, and eGFR in Chinese CKD children for the first time. Our findings emphasize the importance of early monitoring serum uric acid levels in the development and progression of CKD in children. Further multicenter, maximus sample, and prospective studies are needed to reveal the actual relationship between hyperuricemia and associated factors in Chinese CKD children.

## Figures and Tables

**Table 1:** Characteristics of the study population by category of serum uric acid.

Characteristic	Total (*n* = 170)	<390 µmol/L (*n* = 52)	390–540 µmol/L (*n* = 62)	>540 µmol/L (*n* = 56)	*p* *
		(<6.5 mg/dL)	(6.5–9 mg/dL)	(>9.0 mg/dL)	
Age (years)	9.79 ± 4.10	9.44 ± 4.44	9.66 ± 4.12	10.24 ± 3.75	0.696
Boys (%)	99 (58.23)	25 (48.08)	38 (61.29)	36 (64.29)	0.193
BMI (kg/m^2^)	17.34 ± 4.49	16.04 ( 14.69, 18.66)	15.88 (14.47, 19.19)	16.58 (14.57, 19.58)	0.589
SBP (mmHg)	117.81 ± 19.96	115.83 ± 21.48	116.39 ± 18.20	121.21 ± 20.29	0.287
DBP (mmHg)	74.69 ± 17.88	74.35 ± 19.45	72.98 ± 15.83	76.91 ± 18.58	0.501
Hb (g/L)	105.31 ± 26.23	114.62 ± 23.09	104.77 ± 25.29	97.27 ± 27.60	0.002
Alb (g/L)	37.97 ± 8.31	42.35 (36.53, 45.95)	38.10 (31.78, 42.48)	38.75 (34.18, 42.68)	0.047
TC (mmol/L)	5.32 ± 2.56	4.35 (3.40, 5.42)	4.72 (3.99, 6.15)	4.95 (4.11, 6.23)	0.127
TG (mmol/L)	2.37 ± 1.89	1.50 (0.87, 2.67)	2.07 (1.14, 3.83)	2.00 (1.40, 2.92)	0.053
BUN (mmol/L)	12.53 (7.69, 26.13)	8.20 (5.50, 10.92)	12.92 (7.87, 24.32)	25.45 (13.24, 38.65)	<0.001
Cys C (mg/L)	3.09 ± 1.84	1.46 (1.16, 2.71)	2.61 (1.49, 4.24)	4.11 (2.36, 5.43)	<0.001
Scr (µmol/L)	162.50 (95.75, 471.50)	88.00 (73.50, 170.00)	165.00 (102.50, 415.75)	411.00 (142.75, 717.00)	<0.001
eGFR (mL/min/1.73 m^2^)	38.26 (12.68, 66.91)	64.87 (29.91, 90.62)	38.63 (13.24, 66.75)	16.57 (9.22, 38.83)	<0.001
Fib (g/L)	2.97 ± 1.13	2.47 (1.98, 3.26)	2.88 (2.32, 3.57)	2.84 (2.34, 3.67)	0.156
DD (mg/L)	0.96 ± 1.54	0.41 (0.22, 0.90)	0.41 (0.20, 0.94)	0.76 (0.38, 1.51)	0.024
24HUPr (mg/m^2^/d)	690.04 (178.94, 1710.18)	547.41 (77.56, 1395.79)	738.69 (248.49, 2058.53)	673.16 (194.90,1663.15)	0.302
CAKUT *n* (%)	68 (40.00)	19 (36.54)	22 (35.48)	27 (48.21)	0.307
Obesity *n* (%)	14 (8.24)	5 (9.62)	6 (9.68)	3 (5.36)	0.633
Hypertension *n* (%)	95 (55.89)	25 (48.08)	33 (53.22)	37 (66.07)	0.148
Anemia *n* (%)	103 (60.59)	27 (51.92)	36 (58.06)	39 (69.64)	0.215
Short stature *n* (%)	41 (24.12)	13 (25.00)	15 (24.19)	13 (23.21)	0.977
Renal osteopathy *n* ( %)	36 (21.18)	7 (13.46)	14 (22.58)	15 (26.79)	0.225

Abbreviations: BMI body mass index, SBP systolic blood pressure, DBP diastolic blood pressure, Hb hemoglobin, Alb albumin, TC total cholesterol, TG triglycerides, BUN blood urea nitrogen, SUA serum uric acid, Cys C cystatin C, Scr serum creatinine, eGFR estimated glomerular filtration rate, Fib fibrinogen, DD D-Dimer, 24HUPr 24 h urine protein quantity, CAKUT congenital anomalies of the kidney and urinary tract. * Among three groups (<390 µmol/L, 390–540 µmol/L, and >540 µmol/L), the univariate analysis of variance was used for the parametric distribution data or a Kruskal–Wallis test. Bonferroni test was used for categorical data and proportions.

**Table 2:** Correlations between SUA and associated factors for CKD.

Characteristics	SUA (*n* = 170)	
	r	*p*
Age (years)	−0.056	0.464
BMI (kg/m^2^)	−0.067	0.384
SBP (mmHg)	0.128	0.096
DBP (mmHg)	0.05	0.515
Hb (g/L)	−0.281	<0.001
Alb (g/L)	−0.148	0.053
TC (mmol/L)	0.161	0.036
TG (mmol/L)	0.208	0.007
Scr (µmol/L)	0.515	<0.001
BUN (mmol/L)	0.558	<0.001
Cys C (mg/L)	0.488	<0.001
Fib (g/L)	0.051	0.506
DD (mg/L)	0.214	0.005
eGFR (mL/min/1.73 m^2^)	−0.442	<0.001
24HUPr (mg/m^2^/d)	0.089	0.247

Abbreviations: BMI body mass index, SBP systolic blood pressure, DBP diastolic blood pressure, Hb hemoglobin, Alb albumin, TC total cholesterol, TG triglycerides, BUN blood urea nitrogen, SUA serum uric acid, Cys C cystatin C, Scr serum creatinine, eGFR estimated glomerular filtration rate, Fib fibrinogen, DD D-Dimer, 24HUPr 24 h urine protein quantity.

**Table 3:** Results of multiple logistic regression analysis between elevated SUA and associated factors.

Characteristics	Elevated SUA (OR, 95% CI)	*p*
Sex (boys vs. girls)	2.122 (0.917, 4.914)	0.079
Age (years)	1.060 (0.959, 1.172)	0.251
BMI > 95th percentile (vs. ≤95th)	0.947 (0.235, 3.907)	0.938
CAKUT	1.540 (0.660, 3.594)	0.318
Hypertension	0.978 (0.441, 2.169)	0.957
Anemia	3.619 (1.322, 9.905)	0.012
Elevated TC	1.814 (0.719, 4.578)	0.207
Elevated TG	0.697 (0.272, 1.790)	0.453
BUN	1.113 (1.032, 1.200)	0.006
Cys C	0.918 (0.579, 1.456)	0.716
eGFR (mL/min/1.73 m^2^)		
<60 (reference)	1	
60–89	0.386 (0.125, 1.195)	0.099
≥90	0.116 (0.025, 0.532)	0.006

Abbreviations: BMI body mass index, CAKUT congenital anomalies of the kidney and urinary tract, TC total cholesterol, TG triglycerides, BUN blood urea nitrogen, SUA serum uric acid, Cys C cystatin C, eGFR estimated glomerular filtration rate.

## Data Availability

The data sets used and/or analyzed during the current study are available from the corresponding author on reasonable request.
